# Recombinant ESAT-6-CFP10 Fusion Protein Induction of Th1/Th2 Cytokines and FoxP3 Expressing Treg Cells in Pulmonary TB

**DOI:** 10.1371/journal.pone.0068121

**Published:** 2013-06-27

**Authors:** Dolly Jackson-Sillah, Jacqueline M. Cliff, Gloria Ivy Mensah, Emmanuel Dickson, Sandra Sowah, John K A. Tetteh, Kwasi K. Addo, Tom H. M. Ottenhoff, Graham Bothamley, Hazel M. Dockrell

**Affiliations:** 1 Faculty of Infectious and Tropical Diseases, London School of Hygiene & Tropical Medicine (LSHTM), London, United Kingdom; 2 Department of Infectious Diseases, Leiden University Medical Centre, Leiden, The Netherlands; 3 Noguchi Memorial Institute for Medical Research (NMIMR), Accra, Ghana; 4 Respiratory Disease Department, Homerton University Hospital, London, United Kingdom; Colorado State University, United States of America

## Abstract

**Background:**

Early secretory antigenic target 6 (ESAT-6) and culture filtrate protein 10 (CFP-10) are *Mycobacterium tuberculosis* (*Mtb*)–specific antigens that are secreted by actively metabolising bacteria and contribute to the virulence of the bacteria. Their ability to induce Treg and Th2 responses, particularly during the first two weeks of treatment, has not been comprehensively examined to date. The purpose of this work was to characterise Th1, Th2 and Treg responses to rESAT-6-CFP10 fusion protein in TB patients before and during the intensive phase of treatment and in healthy *M.bovis* BCG vaccinated donors.

**Methods:**

Forty-six newly diagnosed, HIV-negative, smear-positive pulmonary TB patients and 20 healthy donors were recruited in the UK and Ghana. Their peripheral blood mononuclear cells (PBMC) were used in *ex vivo* ELISPOT and *in vitro* cultures to identify immunological parameters of interest.

**Results:**

The study confirmed that protective immune responses to rESAT-6-CFP10 are impaired in active TB but improved during treatment: circulating antigen-specific IL-4-producing T-cells were increased in untreated TB but declined by two weeks of treatment while the circulating antigen-specific IFN-γ producing T cells which showed a transient rise at one week of treatment, persisted at baseline levels at two months of treatment. *In vitro* T cell proliferation and IFN-γ production were reduced, while IL-4 and CD4^+^FoxP3^+^CD25^hi^ cell expression were increased in response to rESAT-6-CFP10 fusion protein in untreated TB. These responses were reversed during early treatment of TB.

**Conclusions:**

These observations support further investigations into the possible utility of these parameters as markers of active disease and favourable treatment outcomes.

## Introduction

ESAT-6 and CFP-10 are dominant gamma interferon (IFN-γ)-inducing antigens of live and actively metabolising *Mtb*. They are low molecular weight proteins, encoded by the RD1 (Region of Difference) genomic region of virulent strains of *Mtb* which is missing from *Mycobacterium bovis* BCG,[1]–[3] and are therefore ideal for differentiating between *Mtb*-infected and *M.bovis* BCG-vaccinated individuals. As the RD1 region is associated with virulence of *Mtb* [4], [5] ESAT-6 and CFP-10 may be implicated as virulence factors. In fact ESAT-6 has been found to induce IL-1β production in dendritic cells to direct Th2 differentiation [6]. CFP-10 is been found to markedly lower B7.1 expression on the surface of macrophages and limit the production of free nitrogen and oxygen radicals which are essential for the elimination of mycobacteria [7]. In combination they also play a role in preventing phagosomal fusion and inhibit lipopolysaccharide-induced macrophage activation by down regulating reactive oxidative species [8], [9].

ESAT-6 and CFP-10 are effective stimulators of T cells. Immunoassays for IFN-γ production (IFN-gamma release assays, IGRAs) in response to ESAT-6 and CFP-10 have been performed in TB cases and their contacts in endemic and non- endemic settings. They have been shown in recent meta-analyses to have a pooled sensitivity of ∼80% [10]–[12] and a higher positive predictive value for progression to active TB in latently infected individuals when compared to the tuberculin skin test [13]. Their specificity is increased while the sensitivity is low in a study conducted in an endemic setting [14]. As these antigens are extracellular proteins and secreted by the live intracellular bacteria, they are believed to be partially responsible for the efficacy of live vaccines [15]–[17].

Although these protiens are important in our quest for adequate TB control, the exact nature and extent of the immunological responses they induce have not yet been fully evaluated. Many studies on the immune responses induced by the rESAT-6-CFP10 fusion protein in humans have only focused on IFN-γ release and have not examined the possibility of concomitant induction of type 2 cytokines associated with active disease. In addition, information on sequential immunological changes in TB patients during the first few weeks of treatment when a majority of the live and metabolically active bacteria is eliminated by chemotherapy is generally scanty.

The present study seeks to examine a more comprehensive immunological response to rESAT-6-CFP10 fusion protein in TB patients undergoing intensive chemotherapy and in healthy volunteers with no known history of *Mtb* exposure. This work was based on the hypothesis that RD1 antigens induce a mixed type cytokine and natural Treg responses in active TB which reverses with effective treatment. We have analysed the Th1/Th2 cytokine expression profile of circulating and in vitro generated RD1-specific T cells, assessed their rate of proliferation and eualuated the induction of naturally occuring Treg cells by RD1 antigens.

## Materials and Methods

### Participants

A longitudinal study in which active pulmonary TB patients were followed up at intervals within the first two months of chemotherapy for tuberculosis was conducted. The participants recruited were adults, 16 years of age and above and newly diagnosed with smear positive pulmonary tuberculosis at the Homerton University Teaching Hospital or University College London Hospital between January 2006 and November 2007 and 3 National TB Control Polyclinics in Ghana between May and September 2011. HIV positive and extra-pulmonary TB patients were excluded. The standard chemotherapy for non-drug resistant cases consisted of isoniazid and rifampicin daily for 6 months plus pyrazinamide and ethambutol daily for the first 2 months. Information on clinical features, radiological findings (the presence and distribution of parenchymal abnormalities consistent with infiltrates and/or cavities), sputum acid-fast bacilli counts and cultures were obtained from hospital records.

As controls, healthy subjects were recruited through the London School of Hygiene & Tropical Medicine (LSHTM) Volunteer Blood Donation Scheme. The majority of these volunteers had received *M.bovis* BCG vaccination.

Blood samples (30 ml) were taken from TB patients at the start of treatment, T0 (<5days of treatment) and after a further 1 week (T1), 2 weeks (T2) and 2 months of chemotherapy (T3). Ten of the controls were bled at similar intervals as the cases, to assess random changes in immune responses occurring over time in healthy individuals. Information on demographic characteristics such as age, sex, ethnicity, BCG vaccination history, past history of tuberculosis and time spent in highly TB endemic areas, were obtained from hospital and LSHTM volunteer records.

### Ex-Vivo ELISPOT Assays

Procedures for IFN-γ ELISPOT have been previously described [18]. In this study freshly isolated PBMCs from healthy donors and TB patients were added at 2.5×10^5^ cells/well in a volume of 100 µl in duplicate wells. The antigen used was rESAT-6-CFP10 fusion protein with endotoxin levels below the limit of detection of 50IU/mg, (courtesy K.L.M.C Franken, Department of Infectious Diseases, Leiden University Medical Centre), and was added at 100 µl/well at a final concentration of 5 µg/ml in duplicate wells. Negative control wells had no antigens. Phytohaemagglutinin (PHA), from Sigma (L-9132) was used as the positive control at 5 µg/ml. Plates were incubated overnight for IFN-γ or 48 hours for IL-4 at 37°C, in 5% CO_2_. Spot forming cells (SFCs) were counted with an AID Diagnostika ELISPOT counter. Responses were considered significant if a minimum of 10 SFCs were present per well and were at least 2× that of negative control wells.

### Cell Culture

A 500 µl aliquot of cell suspension containing 10^6^ freshly separated PBMC in RPMI 1640 medium, 10% autologous plasma and 1% L-glutamine (Gibco, lot no-3094131) was placed per well in a 24 well Nunclon plates (VWR: 402/0323/14) under sterile conditions. Antigen preparations (rESAT-6-CFP10 fusion protein) and PHA as a positive control were added to designated wells in duplicate at final concentrations of 5 µg/ml. Plates were incubated for 6 days at 37°C in 5%CO_2_ incubator.

### Flow Cytometric Staining for Cell Surface Markers and Intracellular Cytokines

For the last 3 hours of the incubation, 10 µg/ml of Brefeldin A (Sigma, UK) was added per well to the PBMC cultures to inhibit secretion of cytokines. This step was omitted for Treg staining. At the end of incubation, cells were harvested into 5 ml polystyrene round-bottom Falcon ™ tubes (BD Biosciences, UK) washed and then re-suspended in 100 µl of cold FACS buffer (filter sterilised PBS with 1% Heat inactivated -FCS and 0.1% NaN_3_). To the cell suspension, 5 µl of FITC-labelled monoclonal antibody for CD4 (556615), anti-CD3 PerCP (552851) and anti-CD8 APC (555369) from Pharmingen UK were added and incubated for 30 minutes in the dark at 4°C. Matching fluorescent-labelled isotype control antibody staining for control tubes and single antibody staining for compensation were also performed. After incubation, the cells were washed in FACs buffer and resuspended in 300 µl of 2% paraformaldehyde in PBS as fixation medium and stored at 4°C in the dark for 15 minutes. Cells were washed again in FACS buffer, re-suspended in 100 µl of permeabilisation buffer (PBS, 1%FCS, 0.1%NaN_3_ and 1% saponin pH 7.5) and incubated for 30 minutes with 1 µl PE labelled anti-human IFN-γ and IL-4 or isotype matched control antibodies (PE labelled mouse IgG_1_ and rat IgG_1_ all from BD Pharmingen 554552 and 553924 respectively). For Treg staining, freshly isolated or cultured PBMC were stained with anti-CD3-PE, anti-CD4-PECy7 and anti-CD25-FITC (all from BD Pharmingen, UK) surface antibodies as described above and then with 5 µl of anti-FoxP3-APC (eBiosciences, clone PCH101, cat.4776) for detection of intracellular FoxP3.

Finally, the cells were washed with permeabilisation buffer and re-suspended in 200 µl of 2% paraformaldehyde. Flow cytometric acquisition was carried out using a four-colour FACSCalibur™ flow cytometer (BD Bioscience, UK) and CellQuest software (BD, Biosciences, UK). Compensation was determined by analysis of unstained cells and cells stained with different single fluorescent-labelled antibodies. Parameters were set to measure cells within lymphocyte population that was gated according to size and granularity. A minimum of 30,000 events were acquired for surface staining only and up to 100,000 events for intracellular staining. For Treg cells, CD25^+^ cells were distinguished from CD25^hi^ cells (CD25^+^ cells with high fluorescent intensity of ≥10^2^). This distinction was important because CD25 is also expressed by other cell populations and natural Tregs are usually discerned as CD25 ^hi^ in humans [19]. Analysis was performed with FlowJo™ version 5.7.2.

### T-cell Proliferation Assay

Freshly isolated PBMC were labelled with carboxy-fluorescein diacetate, succinimidyl ester (CFDA-SE) (Molecular Probes, UK, V-12883). A two million cell suspension was incubated with 4 ml of pre-warmed PBS containing 5 µM of CFDA-SE for 15 minutes at 37°C. The cells were then re-pelleted by centrifuging and re-suspended in fresh pre-warmed RPMI medium and incubated at 37°C for a further 30 minutes to ensure complete modification of the probe. After a final wash, cells were re-suspended in growth medium. Aliquots of 500 µl cell suspension containing 0.5×10^6^ labelled cells were incubated with antigens as described above. The cells were then harvested for FACs staining with anti-CD8-APC (BD Pharmingen, UK) and anti-CD3-PECy7 (BD Pharmingen, UK) as described above. For analysis of T cell proliferation, the proportion of CD8^+^and CD8**^−^** T cells within the lymphocyte gate was obtained. Various generations of proliferated T cells were defined by gating on CFSE positive events. A reduction in fluorescent intensity of the CFSE with successive cell divisions allowed the different generations of T cells to be distinguished. The proliferative index (PI) which is the sum of the cells in all generations divided by the calculated number of original parent cells was obtained for CD8^+^ and CD8^−^ T cell populations for each culture condition.

### Statistical Analysis

Data were entered into Microsoft Excel files and exported to GraphPad Prism® Version 6.0 for statistical analysis. Longitudinal assessment of TB cases was done by comparing the mean or median response at each follow up time point with the mean or median baseline response. Test for normality was performed with the D’Agostino and Pearson Omnibus normality test. If the data distributions approximated normality, the repeated measures One-way ANOVA with Sidak’s multiple comparison test was used to analyse longitudinal responses in patients with more than 2 samples taken, the paired t-test was used for those with only 2 samples and the unpaired Student’s t-test for the comparison between baseline responses in TB patients and healthy donors. Data with non-Gaussian distributions were analysed using non-parametric tests. Comparisons between cases and healthy controls were done with the Mann-Whitney test. Longitudinal comparison among cases was done with the non-parametric repeated measures one-way ANOVA test (Friedman’s test) with Dunns post test for patients with more than 2 samples and the Wilcoxon matched-pairs signed rank test for those with 2 samples. The correlation between T reg and the ratio of Th1/Th2 responses was analysed with Spearman correlation.

### Ethics Statement

Details of the study were carefully explained to the TB patients and controls, who were asked to read and sign a free and informed consent form approved by the LSHTM and the National Health Service Research Ethics Committees (reference 05/MRE09/8) as well as the IRB of NMIMR (reference IRB00001276).

## Results

### Demographic and Clinical Information

A total of 66 subjects were included in this analysis. Their demographic and clinical information is shown in **Table 1**. Ten patients completed follow up with four blood samples taken, ten had three blood samples taken, twenty four had two blood samples taken and only two individuals had just one sample taken. Twenty six of the 46 patients had a visible BCG scar and those without scars did not have any recollection of BCG vaccination. All patients were sputum smear and culture positive for *Mtb* at diagnosis and achieved sputum conversion by 2 months of treatment.

**Table 1:** Clinical and demographic information on study participants.

Variable	Subcategory	Number (%)
**Participants**		**66 (100)**
**A.TB patients**		**46 (70)** *
Mean age (range)		36 (18–65)
Sex	Male	26 (57)
	Female	20 (43)
Clinical symptoms	Productive cough	42 (91)
	Fever	40 (87)
	Night sweats	20 (43)
	Dyspnoea	10 (22)
	Loss of weight	35 (76)
	Haemoptysis	2 (4)
Chest X-ray	Infiltrates	46 (100)
	Cavitation	28 (61)
	Hilar Lymphadenopathy	10 (22)
Ethnicity	Africans	30 (65)
	Asians	14 (30)
	Caucasians	2 (5)
**B. Healthy donors**		**20 (30)** *
Mean age (range)		30 (24–45)
Sex	Male	4 (20)
	Female	16 (80)
Ethnicity	Caucasians	19 (95)
	Africans	1 (5)

* The percentage of total number of participants.

### Circulating IFN-γ and IL-4 Producing rESAT-6-CFP10-specific T Cells

Ten out of 46 patients were enrolled for ELISPOT assay. All ten patients were bled at baseline (T0), nine 7 days later (T1) and seven 14 days later (T2) and six at 2 months of treatment (T3) due to losses to follow up.

**Figure 1:**
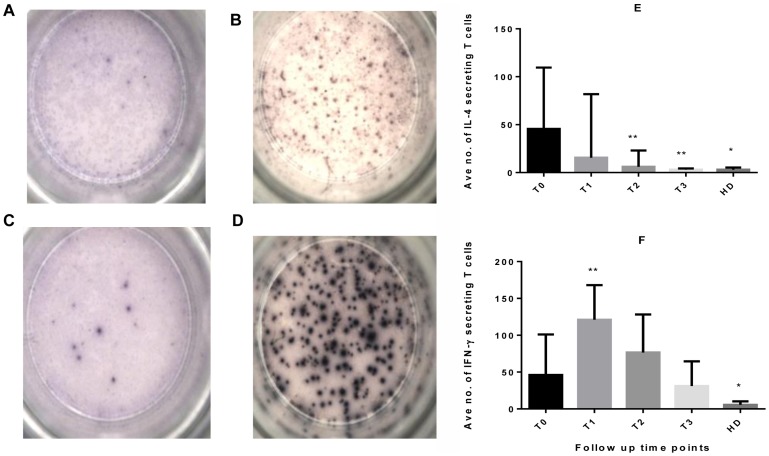
The frequency of IL-4 and IFN-γ secreting cells in response to rESAT-6-CFP10 fusion protein in TB patients and healthy donors. Freshly isolated PBMC from 10 TB patients and 10 healthy donors were used in an ELISPOT assay. The photographs show IL-4 (A & B) and IFN-γ (C & D) ELISPOTS derived from rESAT-6-CFP10 fusion protein stimulated (B & D) or unstimulated (A & C) cells. The frequency of IL-4 (E) and IFN-γ (F) secreting rESAT-6-CFP10-specific T cells in 6 of these patients is shown as the median number of cells per 250,000 PBMC corrected for background responses. Error bars are the inter-quartile range of the median. Statistical comparisons were made between T0 and other time points for TB patients using the non-parametric repeated measures one-way ANOVA (Friedman’s test) with Dunn’s multiple comparison and between T0 and healthy donors using the Mann Whitney test (**P<0.05 in the longitudinal comparison and * P<0.05 in unpaired variables ). *(T0 = baseline, T1 = 1week, T2 = 2weeks T3 = 2months of treatment, HD = healthy donors).*

**Figure 1** shows representative IFN-γ and IL-4 ELISPOT plates and corresponding summary results. Recombinant ESAT-6-CFP10-specific IL-4 secreting cells were present in all patients at diagnosis (median SFCs of 45 cells/250,000 PBMC). This reduced significantly two weeks later (P = 0.0097). By 2 months of treatment there was no detectable response to IL-4 (P = 0.0001, **Figure 1E**). IL-4 in healthy donors remained significantly lower than baseline values for TB patients (P = 0.0002).

Circulating rESAT-6-CFP-10-specific IFN-γ secreting T cells were present in all patients at diagnosis of TB. This significantly increased in frequency from median SFCs of 46 cells/250,000 PBMC at baseline to 121 cells/250,000 PBMC one week later and reduced by 2 months of treatment to the frequency seen at baseline. None of the patients achieved a complete reversion in IFN-γ response by two months of treatment. Responses were significantly lower in healthy donors compared to TB patients (**Figure 1F**).

### ESAT-6-CFP10-induced in vitro Expression of IFN-γ and IL-4 Producing T Cells

**Figure 2:**
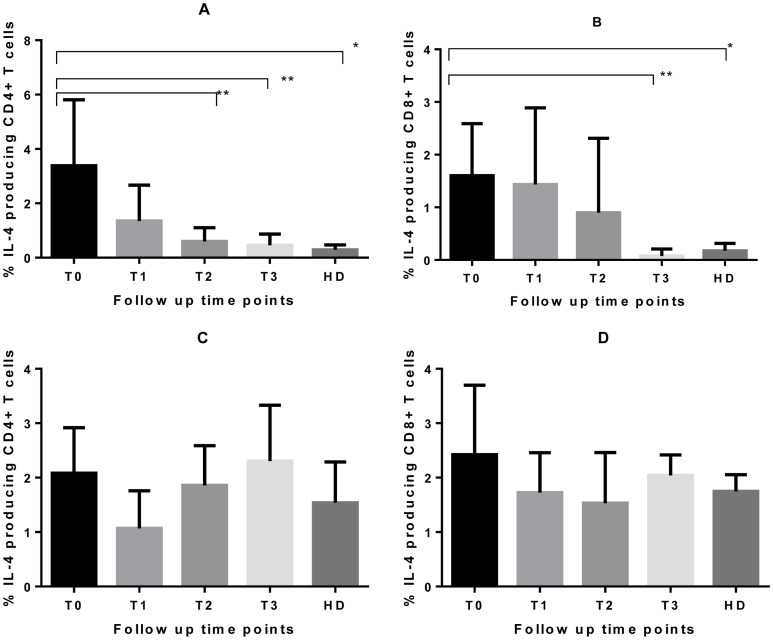
rESAT-6-CFP10 induced IL-4 responses in tuberculosis patients and healthy*M.bovis* BCG vaccinated healthy donors. PBMC obtained from 10 active TB patients and 17 healthy donors were cultured in the presence of rESAT-6-CFP10 or PHA for 6 days. Cells were then stained to detect the presence of intracellular IL-4. The figure represents the overall summary for rESAT-6-CFP10 (A & B) and PHA responses (C & D) in CD4^+^ (A & C) and CD8^+^ (B & D) T cells. The bars represent the means and the error bars are 95% CI. P-values were obtained from the Mann-Whitney test for comparison of healthy donors with T0 and comparisons were made between T0 and other time points for TB patients using the non-parametric repeated measures one-way ANOVA (Friedman’s test) with Dunn’s multiple comparison (**P<0.05 in longitudinal comparison and * P<0.05 in unpaired variables ). Abbreviations are described in **Figure 1**.

To identify specific T cells producing IFN-γ and IL-4 in response to RD1 antigens in vitro, PBMC from TB patients and controls, cultured for 6 days in the presence of rESAT-6-CFP10 fusion protein, were harvested and stained for intracellular expression of IL-4 (N = 19, 10 had a complete set of follow up data, and 9 had only baseline and two weeks data ) and IFN-γ (N = 39, 10 had a complete set of data, 22 had baseline and two weeks, 4 had baseline, two weeks and two months, 2 had baseline and one week and 1 individual had only baseline data. The last three individuals were excluded from the longitudinal analysis because of insufficient numbers). For patients with a complete set of data (**Figure 2** **&** **3**), the mean proportion of CD4^+^ T cells expressing IL-4 (**Figure 2A**) was significantly higher at diagnosis compared to two weeks (P = 0.0071) and two months later (P = 0.005). A later reduction in IL-4 expression was observed at 2 months of treatment in CD8^+^ T cells (**Figure 2A &B**). A similar finding was observed in patients who only had the baseline and week two samples analysed (Wilcoxon P-value = 0.0234 for CD4^+^ but 0.2367 for CD8^+^ T cells; data not shown). The mean frequency of IL-4 expressing T cells in healthy donors was much lower compared to TB patients at diagnosis (Mann Whitney P-value = 0.0008 for CD4^+^ and 0.0003 for CD8^+^T cells). IL-4 producing CD4^+^ and CD8^+^ T cells in TB patients were the same as in healthy donors by two weeks and two months of treatment respectively.

**Figure 3:**
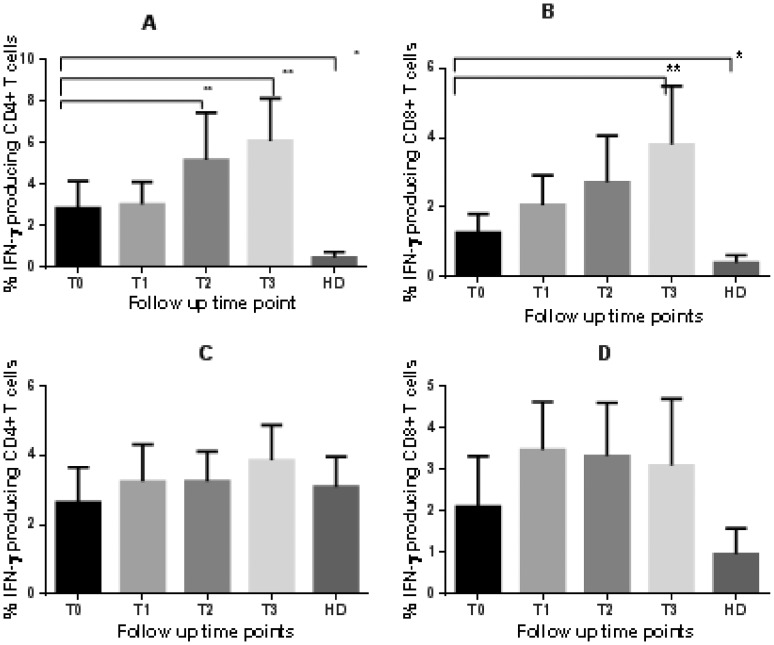
ESAT-6/CFP-10 induced IFN-γ responses in tuberculosis patients and healthy *M.bovis* BCG vaccinated donors. PBMC obtained from 10 active TB patients and 17 healthy donors were cultured in the presence of ESAT-6/CFP-10 and PHA for 6 days. Cells were then stained to detect the presence of intracellular IFN-γ. The figure represents the overall summary of ESAT-6/CFP-10 (A & B) and PHA responses (C & D) in CD4^+^ (A &C) and CD8^+^ (B &D) T cells. The bars represent the means and the error bars are 95% CI. P-values were obtained from the unpaired t-test for comparison of healthy donors with untreated TB patients and the repeated measures one-way ANOVA with Sidak’s multiple comparison test was used for comparison of longitudinal data with the baseline results in patients (**P<0.05 in longitudinal comparison and * P<0.05 in unpaired variables). Abbreviations are described in **Figure 1**.

IFN-γ expression increased significantly from the baseline to two weeks in CD4^+^ T cells (**Figure 3A** **)**. However data analysis of the 22 patients with only baseline and week two results suggested a statistically significant increase in responses in both CD4^+^ (Wilcoxon P = 0.0016) and CD8^+^ (P = 0.0004) T cells (data not shown). An increase was observed at two months of treatment in both CD4^+^ and CD8^+^ T cells (**Figure 3B**). Again, responses in healthy donors were lower compared to TB patients at diagnosis (unpaired Student’s t-test P = 0.0007 for CD4^+^ and 0.0027 for CD8^+^ T cells).

**Figure 4:**
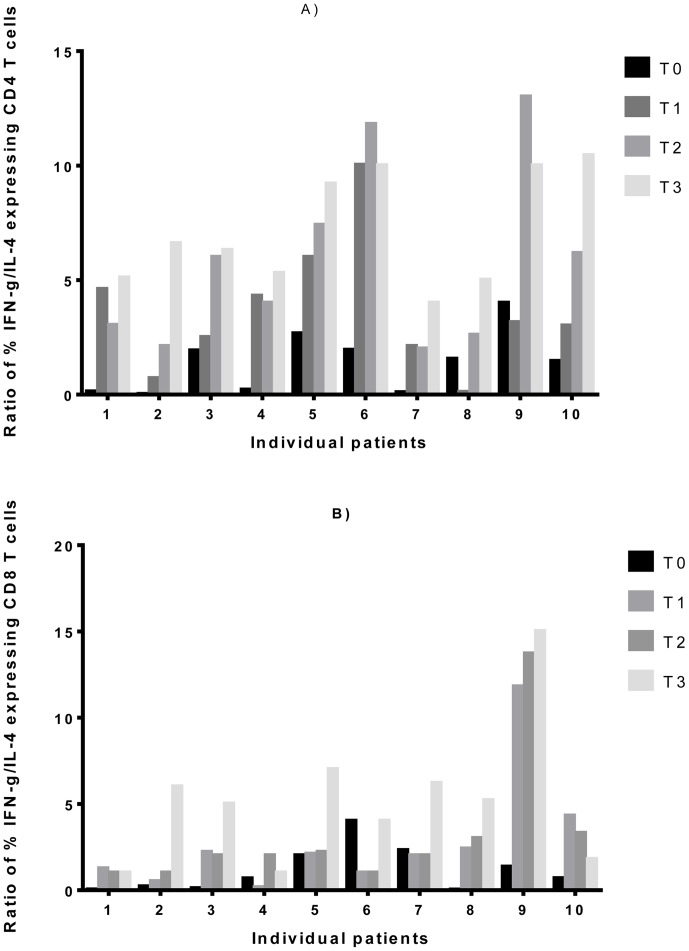
rESAT-6-CFP10-induced changes in the ratio of the proportion of T cells expressing IFN-γ/IL-4 in tuberculosis patients. PBMC obtained from 10 active TB patients and were cultured in the presence of rESAT-6-CFP10. Cells were then stained to detect the presence of intracellular IFN-γ and IL-4. The ratio of the proportion of CD4^+^ (A) and CD8^+^ (B) T cells expressing IFN-γ/IL-4 for individual patients at diagnosis and during follow up are represented. The significant p-values obtained from the Friedman’s test with Dunn’s post-test correction for the mean ratios were 0.0017 at T2 v T0 and 0.001 at T3 v T0 in CD4^+^ T cells and 0.0022 at T3 v T0 in CD8^+^ T cells. Abbreviations are described in **Figure 1**.

The ratio of IFN-γ/IL-4 expressing T cells at diagnosis of TB increased progressively from 2 weeks after initiation of treatment in CD4^+^ T cells (P = 0.0097) and by two months of treatment in CD8^+^ T cells (P = 0.0022): results obtained from individual patients are illustrated in **Figure 4**. At diagnosis, these patients were smear positive, had pulmonary infiltrates and 5 had pulmonary cavitation. All patients achieved sputum conversion at two months of treatment. Despite the similarity in clinical presentation, there was a substantial individual variation in responses particularly with the CD4^+^ T cell ratios which did not seem to be related to the presence or absence of pulmonary cavities.

### T cell Proliferative Responses to rESAT-6-CFP10 Fusion Protein

**Figure 5:**
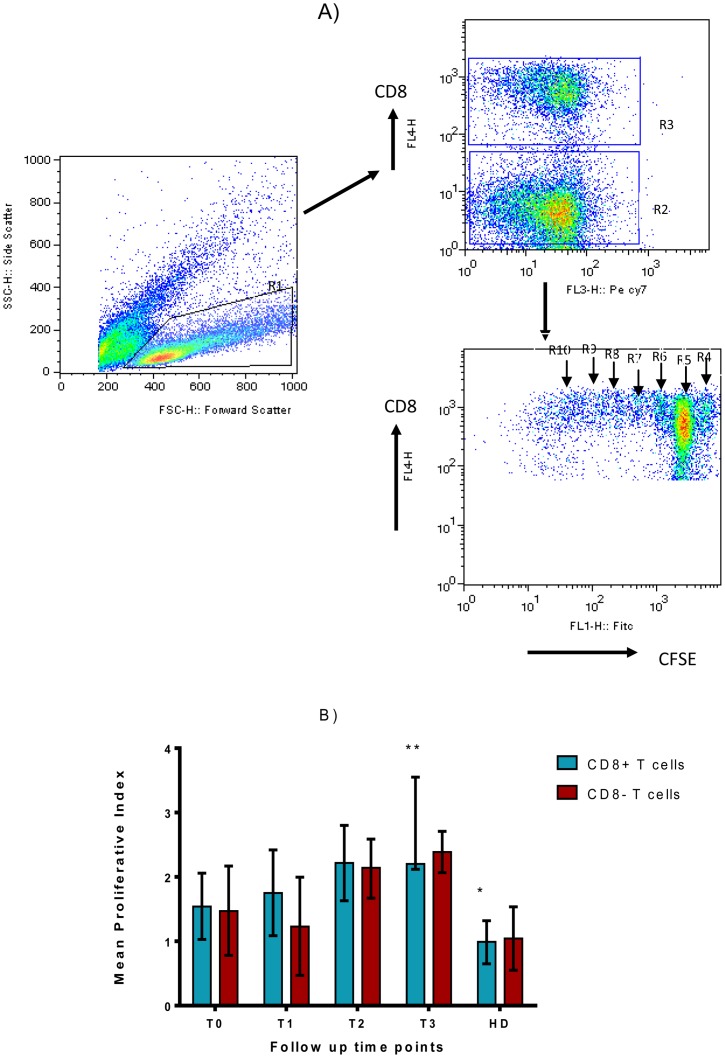
Gating strategy and results of T cell proliferation assay. Freshly isolated PBMC from 10 TB patients and 10 healthy donors were stained with 5 µM of CFDA-SE and then cultured in the presence of rESAT-6-CFP10 fusion protein (5 µg/ml), PHA (5 µg/ml) or no antigen for 6 days. Harvested cells were stained with anti-CD8-PECy7. Live lymphocytes were gated using the forward and side scatter characteristics to include both resting and dividing cells (R1). CD8+ and CD8- Cells (R3 and R2) were then obtained from the lymphocyte gate. CFSE staining in each cell population was obtained and the different generation of cells were gated R4 to R10. The **Figure 5A** is representative sample from a control subject. With reference to **Figure 5A** above, T cell proliferation for 10 TB patients is shown (**Figure 5B**). The bars represent the mean PI (± SE) for CD8^+^ and CD8^−^ T cells after subtracting the baseline proliferation. Abbreviations are described in **Figure 1**.

A reduction in fluorescence intensity of CFSE with successive cell division allows different generations of T cells to be distinguished and gated as shown in **Figure 5A**. rESAT-6-CFP10 fusion protein-induced proliferative responses in CD8^+^ and CD8^−^ T cells were lower at diagnosis of TB but increased slightly during follow up. This was only statistically significant in CD8^+^ T cells by two months of treatment (**Figure 5B**). This indicates that the rESAT-6-CFP10 fusion protein-specific precursor T cell proliferation rate increased during treatment of TB and this is particularly evident in CD8^+^T cells. In CD8^+^ T cells PI values obtained for healthy donors were significantly lower than that for TB patients at diagnosis (Mann Whitney P-value = 0.0131).

### Circulating CD25^+^Foxp3^+^CD4^+^ T Cells in Active TB

The frequency of circulating CD25^+^FoxP3^+^CD4^+^ or CD25^+^FoxP3^+^CD4^−^T cells was assessed in 26 TB cases (10 had a complete data set, 10 had baseline and two weeks, 4 had baseline, two weeks and 2 months, and 2 had baseline and one week data; the last two groups of patients were excluded from the longitudinal analysis because of insufficient numbers) and 20 healthy donors. The expression of CD25^+^FoxP3^+^CD4^+^ T cells was much higher in frequency in TB patients at diagnosis compared to the healthy donors (mean ± SD of 1.92±1.05% compared to 0.88±0.36%, P = 0.032). This reduced significantly by 2 months of treatment (1.18±0.12% P = 0.0450) but not after one or two weeks of treatment. FoxP3 expression was also detected in CD25^−^CD4^+^ T cells (data not shown).

### ESAT-6-CFP10 Induction of CD25^+^FoxP3^+^CD4^+^ T Cells

**Figure 6:**
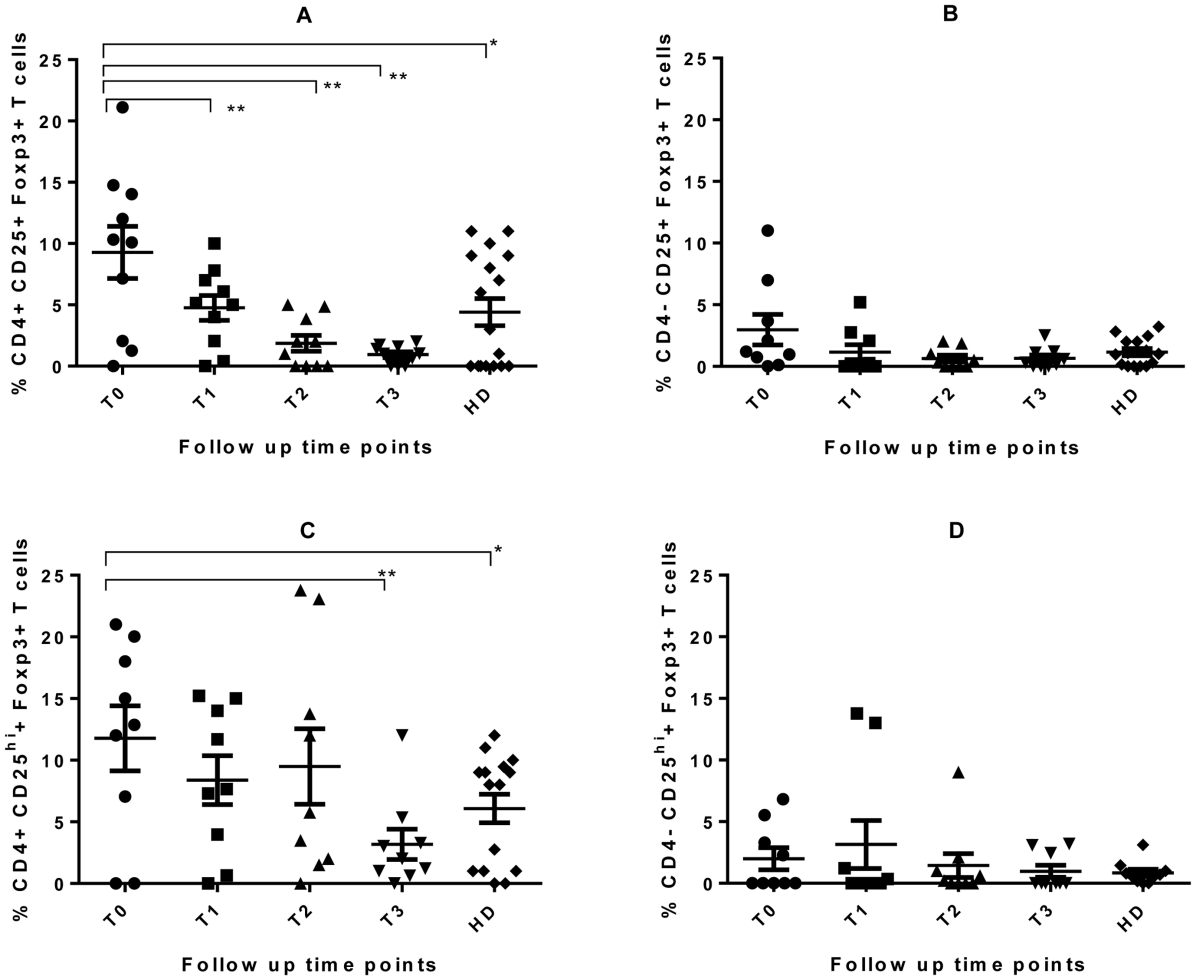
rESAT-6-CFP10-induced regulatory T cells in TB patients and healthy donors. Freshly isolated PBMC from 10 TB patients were cultured in the presence of ESAT-6-CFP10 or without antigen for 6 days. Harvested cells were then stained for the expression of CD25 and Foxp3 within the CD4^−^ (B & D) and CD4^+^ (A & C) T cell populations. The figure illustrates the mean proportions (horizontal lines) of CD4^+^ and CD4^−^ T cells expressing both CD25 and FoxP3 (A & B) or CD25 with high fluorescent intensity (CD25^hi^) and Foxp3 (C & D) after subtracting the responses obtained in unstimulated cultures. The dots are the individual responses and the error bars represent the standard errors of the mean. The repeated measures one-way ANOVA with Sidak’s multiple comparison tests was used for comparison of longitudinal data with the baseline results in patients. The Mann Whitney p-value was used in the comparison of baseline results from TB patients with healthy donors (**P<0.05 in longitudinal comparison and * P<0.05 in unpaired variables) Abbreviations are described in **Figure 1**.

**Figure 7:**
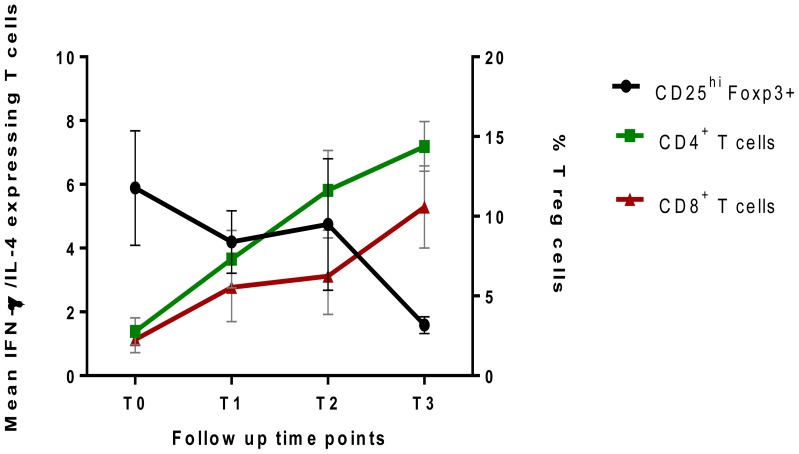
An illustration of the trend in rESAT-6-CFP10-induced regulatory T cell expression and IFN-γ/IL-4 ratios in the intensive phase of TB treatment. Freshly isolated PBMC from 10 TB patients were cultured in the presence of ESAT-6-CFP10 or without antigen for 6 days. Harvested cells were then stained for the expression of CD25, Foxp3, IFN-γ and IL-4. The mean regulatory T cell expression is plotted against the mean of the ratio of IFN-γ/IL-4 expressing T cells at diagnosis and during follow up of TB patients. Abbreviations are explained in **Figure 1**.

**Figure 6** illustrates the in vitro T reg frequencies after a 6 day culture of PBMC from 10 TB patients and 15 healthy donors with rESAT-6-CFP10 fusion protein. The expression of FoxP3 by CD25^+^CD4^+^ T cells was high after antigen stimulation of cells obtained from patients before treatment. The mean baseline frequency of 9.3±2.0% reduced gradually to 0.94±0.16% by 2 months of treatment (P = 0.0067) and was higher than the mean frequency of 4.4±1.1% observed in healthy donors (**Figure 6A**). This reduction in Treg frequency was detected as early as one week of treatment (P = 0.0109). The CD4^−^FoxP3^+^CD25^+^ T cell frequency was observed to be much lower than the CD4^+^ counterpart and frequencies remained the same during follow up of TB cases and were similar to levels in healthy donors (**Figure 6B**). The expression of Foxp3 by CD25^hi^CD4^+^ showed a significant reduction by 2 months of treatment while the expression by CD25^hi^CD4^−^ remained the same (**Figure 6C & D**). **Figure 7** shows a decline in the mean regulatory T cell frequency with an increasing mean IFN-γ/IL-4 cytokine ratio in both CD4^+^ and CD8^+^ T cells during the intensive phase of TB treatment. A significant negative correlation between CD25^hi^ FoxP3^+^ CD4^+^ T cells and IFN-γ/IL-4 cytokine ratios was observed for CD4^+^ T cells (Spearman r = -0.47, P = 0.0032) and CD8^+^ T cells (Spearman r = -0.34, P = 0.0378).

## Discussion

Determination of ex vivo and in vitro RD1-specific Th1 and Th2 cytokine expression profile in active TB and their longitudinal tracking during early therapy provides valuable insights into immune responses induced by the antigen in the pathogenesis of TB. This could aid the identification of possible biomarkers for disease activity and early treatment response.

Our results demonstrated the presence of endogenous IL-4 and IFN-γ secreting rESAT-6-CFP10-specific T cells in untreated active TB. While the circulating IFN-γ secreting rESAT-6-CFP10-specific T cells persisted, IL-4 secreting T cells disappeared within two weeks to two months of treatment. Recombinant ESAT-6-CFP10 fusion protein also induced higher in vitro levels of Tregs and IL-4 expressing T cells in untreated TB compared to two weeks to two months after initiation of treatment. Conversely, the antigen induced lower in vitro levels of IFN-γ expressing T Cells in untreated TB compared to two weeks to two months after initiation of treatment, resulting in an increasing IFN-γ/IL-4 T cell ratio during the follow up period. The initial impairment of T cell proliferation observed in untreated TB also improved in the first two months of treatment, particularly for CD8^+^ T cells.

The identification of circulating IFN-γ and IL-4-producing RD1-specific T cells ex vivo, as measured in the ELISPOT assay, indicate a recent encounter with antigen from *Mtb* in vivo and their overall frequencies may be directly related to bacterial load in patients undergoing treatment for pulmonary TB. This has been suggested previously for IFN-γ (but not IL-4) specific cells [20] and raises the possibility that the ELISPOT test could be used to monitor response to treatment.

The RD1-specific IFN-γ producing T cell frequency in the present study increased by one week of treatment and declined by 2 months of treatment to baseline levels, maintaining a circulating pool of effector/effector memory T cells. Similar longitudinal examination of IFN-γ responses in TB patients using the T-SPOT TB® and QuantiFERON®-TB Gold tests showed some decline in responses after therapy with a good number of subjects remaining positive at 6 months after commencing treatment [21]–[23]. These studies however did not examine the first few weeks after initiation of chemotherapy and therefore have not reported an initial increase in response as shown in the present study. The short term increase in antigen-specific IFN-γ producing effector cells in the present study may be the result of an improvement in immune responses after initiation of TB treatment or antigen release after killing of mycobacteria [24]. Failure of complete reversion of IFN-γ release assays after 2 months of treatment and individual variability in responses may limit their utility as a surrogate marker for early treatment efficacy, thus suggesting the need for multiple biomarkers.

The presence of circulating IL-4 secreting RD1-specific T cells in untreated active TB is indicative of *Mtb* antigen-induced suppression of the pro-inflammatory IFN-γ response; a shift towards Th2 bias. This has been demonstrated previously with the use of phorbol myristic acetate (PMA) and Ionomycin as stimulants and flow cytometric detection of the intracellular cytokine [25]. Our result is however in contrast with a recent study which did not detect IL-4 responses in TB patients using the ELISPOT assay [26]. One possible explanation is that the patients examined in the latter study had a milder clinical spectrum of disease due to the inclusion of paucibacillary forms such as culture negative pulmonary TB and lymph node TB and were also recruited up to 4 weeks after initiation of therapy. Also PBMC incubation with peptide for the detection of IL-4 specific cells was only done for 14 hours in contrast to 48 hours for the present study. From the present study, the IL-4 release assay may provide a better immunological marker for a favourable treatment response even as early as two weeks after initiation of therapy. A larger study is however required to evaluate the possible utility of this parameter to predict treatment outcomes.

The IFN-γ based ELISPOT assay does not distinguish between latent infection and active TB. In this report, the IL-4 ELISPOT responses were significantly higher in TB patients compared to the *M.bovis* BCG vaccinated healthy donors. Earlier studies identified IL-4 responses in TB contacts that subsequently progressed to clinical disease [27], [28]. Thus assessing the dynamic IL-4 and IFN-γ profiles from endogenously activated T cells may help distinguish between disease progression, resolution and latent infection. A progressive disease may be characterised by high levels of circulating IL-4 and IFN-γ expressing *Mtb*-specific effector/effector memory T cells and disease resolution by the reduction in circulating IL-4 expressing Mtb-specific T cells, while latent infection may be characterised by high levels of IFN-γ expression with little or no IL-4 secreting *Mtb*-specific cells. This hypothesis warrants further validation in a larger cohort of TB cases and latently infected individuals.

To assess the cytokine expression profile of RD1-specific central memory T cells in the pathogenesis of TB, T cell proliferation and differential expression of Th1 and Th2 cytokines by CD4^+^ and CD8^+^ T cells was evaluated in vitro in a 6-day culture assay. The results indicated that in vitro antigen-induced expression of IL-4 secreting T cells was higher at diagnosis than at two weeks to two months of treatment. This is consistent with the reduction in circulating *Mtb*-specific IL-4 secreting effector/effector memory cells in response to the declining bacterial load and also suggests that the improved proliferative responses observed in vitro are not likely to be from the RD1-specific IL-4 precursor cell lineage. The decline in IL-4 producing cells is aligned with sputum conversion indicating that the expression of IL-4 may be associated with the presence of live *Mtb*. In contrast, IFN-γ expressing T cells and the IFN-γ/IL-4 T cell ratios which were lower in untreated active TB, increased in proportion during treatment. This finding is consistent with similar reports that measured ex vivo cytokine mRNA [29] and intracellular cytokine [30]. The increasing in vitro IFN-γ expressing cells may be linked to the increasing proliferative responses observed in CD8^+^ T cells. Surprisingly there was no significant increase in proliferative responses in CD8^−^ T cells. Since these cells are predominantly CD4^+^ T cells, one possible explanation is that their proliferative responses were stifled by the reduction in expansion of CD4^+^ CD25^+^ FoxP3^+^ T cells during TB treatment as we have demonstrated or simply because our sample size did not have enough power to detect any significant differences in proliferation in that population of cells.

The reasons for the impaired *Mtb*-specific T cell function in active tuberculosis remain controversial. Previous reports have suggested a role for CD4^+^CD25^+^Treg cells in tuberculosis [30]–[32]. We have demonstrated that CD4^+^CD25^+^FoxP3^+^T cells are increased in the peripheral blood of active TB patients compared with *M.bovis* BCG vaccinated healthy donors. This finding is consistent with the recently reported CD4^+^CD25^+^FoxP3^+^ Treg responses in humans [30] and in the murine TB model [33], [34]. In addition, the finding that the circulating Treg cells in the peripheral blood declined progressively by 2 months of anti-TB treatment is also in agreement with a recent report on CD4^+^CD25^+^FoxP3^+^ T cells in human tuberculosis [31]. In the present study, a small proportion of circulating CD4^+^CD25^−^T cells and CD4^−^T cells expressed FoxP3. The regulatory role of these cells has been shown in previous studies to be similar to that of the CD4^+^CD25^+^FoxP3^+^ Treg cells in the murine model [35], [36].

The fact that rESAT-6-CFP10 fusion protein induced more CD4^+^CD25^+^Foxp3^+^ or CD4^+^CD25^hi^FoxP3^+^Treg cells at diagnosis of TB than at 2 months of treatment may suggest that the inflammatory response driven by *Mtb* is regulated by naturally-occurring Treg, which are capable of expansion in response to recall antigens. What needs clarification is whether these cells are antigen-specific and expand by proliferating in response to antigen or whether the presence of antigen induces an increased gene expression of Treg phenotypes in naïve T cells destined for regulatory functions. A combination of both theories may be responsible for the findings of this study. Firstly, regulatory T cells induced in vitro reduced in frequency by 2 months treatment suggesting that as the *Mtb* is eliminated, there is minimal induction of Treg cells and subsequent reduction in their precursor frequency as highlighted by the decreasing ex vivo responses. Secondly rESAT-6/CFP-10 induced substantial natural Treg expression *in vitro* in healthy *M.bovis* BCG vaccinated donors and this may support the induction of natural Treg gene expression in naïve T cells in the presence low intensity activation with antigens and absence of inflammation. Another important question therefore is whether the *in vitro* induced FoxP3 Treg cells have similar regulatory functions in human TB and other infectious diseases as the naturally occurring Treg cells. The kinetics of natural Treg expression during intensive treatment of TB followed a similar pattern as the expression of IL-4 by *Mtb*-specific T cells both *in vitro* and *ex vivo*. There was also a significant negative correlation between the in vitro Treg cell frequency and the IFN-γ/IL-4 cytokine expression ratios. It is therefore possible that the functions of the antigen induced CD4^+^CD25^hi^FoxP3^+^ Treg cells in tuberculosis have similar regulatory function as the naturally occurring Treg cells and may be mediated by IL-4 expression. Further functional studies will however be required to confirm or refute this finding.

Culturing CD4^+^CD25^−^FoxP3^+^ Treg cells in the presence of antigens possibly resulted in the generation of CD4^+^CD25^+^FoxP3^+^Tcells because the CD4^+^CD25^−^FoxP3^+^T cell phenotype disappeared after 6 days of culture. Although apoptosis of CD4^+^CD25^−^FoxP3^+^T cells may occur in culture, studies in the murine model of TB have indicated that the CD4^+^CD25^−^Foxp3^+^T cell phenotype is a committed regulatory T cell that regains CD25 expression in vitro after culture with antigen [36]. The CD4^−^ regulatory T cells remained unchanged after culture raising the possibility that they may not have immune regulatory functions in human immune responses. Other markers have been proposed as Treg markers suggesting the heterogeneous nature of these cells [37], [38] and the need to include a variety of markers for their effective isolation and functional characterisation. Irrespective of our choice of Treg marker, the pathogenic significance of our finding is that the high levels of RD1-specific regulatory T cells and the depressed IFN-γ responses in untreated TB suggests inhibition of protective Th1 immune responses, facilitating pathogen multiplication and dissemination. Though suppression of Th1 responses may also reduce inflammation and tissue damage, all our patients had substantial parenchymal infiltrates and over 60% had pulmonary cavities. Perhaps this could be explained by results from a previous study that demonstrated that in TB, Treg cells do not inhibit the proinflammatory Th17 responses, thus enhancing tissue damage through the formation of necrotising granulomas [39].

This study has demonstrated certain aspects of the effector functions of circulating and exogenously generated rESAT-6-CFP10-specific T cells in untreated TB and during the intensive phase of TB treatment. The potential use of the IL-4 ELISPOT assay in monitoring patient response to treatment and differentiating between latent and active TB is described. High proportions of circulating and *in vitro* rESAT-6-CFP10 fusion protein-induced CD4^+^CD25^hi^FoxP3^+^ Treg cells are present in untreated TB patients and may suppress protective immune responses. These results therefore illustrate the complexity of the immune response to a single fusion protein during tuberculosis and the extent to which these responses are modulated rapidly with anti-tuberculosis treatment. Further research with latently infected individuals will shed more light on the nature of the immune responses elicited by rESAT-6-CFP-10 fusion protein and the possible utilisation of some of these parameters to determine disease aggravation and protective immunity.
